# Le syndrome d'insensibilité complète aux androgènes: à propos de deux cas et revue de la literature

**DOI:** 10.11604/pamj.2015.20.400.6760

**Published:** 2015-04-23

**Authors:** Boutaina Lachiri, Ihssane Hakimi, Adil Boudhas, Khalid Guelzim, Jaouad Kouach, Mohamed Oukabli, Driss Moussaoui Rahali, Mohamed Dehayni

**Affiliations:** 1 Service de Gynécologie-obstétrique, Hôpital Militaire d'Instruction Mohammed V, Rabat, Maroc; 2 Service d'Anatomie Pathologique, Hôpital Militaire d'Instruction Mohammed V, Rabat, Maroc

**Keywords:** Syndrome d´insensibilité complète aux androgènes, testicule féminisant, tumeur de Sertoli-Leydig, orchidectomie, Complete androgen insensitivity syndrome, testicular feminization, Sertoli-Leydig tumor, orchidectomy

## Abstract

Le syndrome d'insensibilité complète aux androgènes (SICA) est une entité rare qui correspond à la forme complète des pseudohermaphrodismes androgynoïdes. Son incidence est en fait très variable, allant, selon les auteurs de 1/20000 à 1/60000 naissances. Il est caractérisé par la coexistence chez le même sujet d'un caryotype masculin (46 XY), avec des gonades males, et d'une morphologie féminine normale. Les auteurs rapportent deux observations de deux jeunes filles présentant le SICA ayant consulté pour aménorrhée primaire, illustrant les particularités cliniques, anatomopathologiques et biologiques du syndrome avec certaines particularités.

## Introduction

Le syndrome de résistance complète aux androgènes (SICA) ou anciennement appelé testicule féminisant (TF) est une affection rare qui correspond à la forme complète des pseudohermaphrodismes androgynoïdes. C'est une maladie génétiquement déterminée récessive liée à l'X, en rapport avec des mutations au niveau de Xq11-q12 des gènes du récepteur des androgènes [1]. Elle est caractérisée par un trouble de la réceptivité périphérique aux androgènes secrétés par les testicules. Ces sujets ont un sexe génétique et gonadique masculin, mais phénotypique féminin [2]. Ce syndrome est souvent diagnostiqué au cours de la période pubertaire quand la patiente consulte pour aménorrhée primaire. Le SICA est accompagné d'un développement anormal des testicules et d'un risque élevé des tumeurs malignes des cellules germinales débutant après la puberté. Les tumeurs à cellules de Sertoli-Leydig sont des tumeurs gonadiques très peu répandues, mais elles sont bénignes dans la majorité des cas [1]. Les auteurs rapportent deux observations de deux jeunes filles présentant le SICA illustrant les particularités cliniques, anatomopathologiques et biologiques du syndrome avec certaines particularités.

## Patient et observation

### Observation 1

**Figure 1:**
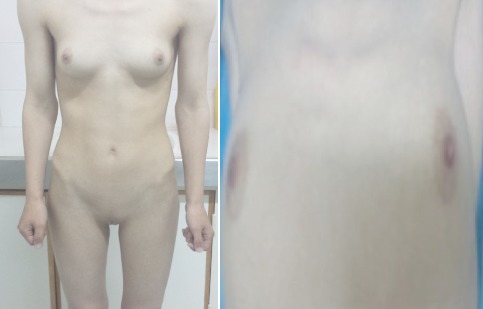
La patiente, morphotype féminin normal mais absence de pilosité pubienne et axillaire

**Figure 2:**
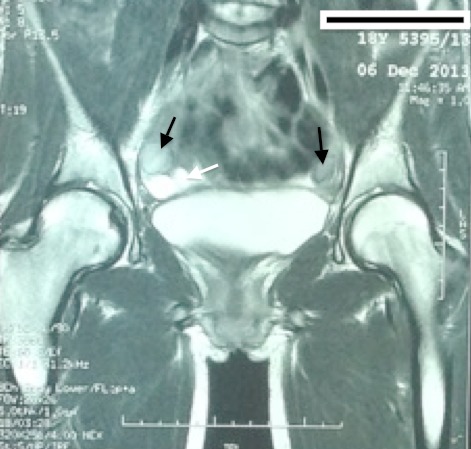
Deux formations oblongues (flèches noires) bilatérales de 44mm de grand axe, tissulaires en discret hypersignal T2 de siège rétro-péritonéal iliaque externe bilatérales avec des formations kystiques polaires inferieures de 17 mm de grand axe (flèche blanche)

O.K. est une jeune fille de 17 ans, issue d'un mariage non consanguin, Troisième d'une fratrie de quatre. Aucune de ses trois sœurs n'a présenté une aménorrhée primaire. Elle a bénéficié à l’âge de 5 ans d'une biopsie d'une tuméfaction inguinale droite dont l'examen histologique a objectivé un testicule immature. Le diagnostic d'un testicule féminisant a été posé et le médecin traitant a expliqué à la famille la nécessité de suivi dans un centre spécialisé mais la famille a démentis les propos énoncés par l’équipe médicale. Vers l’âge de 12 ans, un développement normal des seins est apparu avec un morphotype féminin harmonieux. Une aménorrhée primaire a été le motif de sa consultation à l'unité de gynécologie-obstétrique. L'examen clinique constate une patiente en bon état général, de phénotype parfaitement féminin qui pèse 50 kg pour 1,74 m, soit un indice de masse corporelle à 17,30 kg/ m^2^. Le développement mammaire normal (stade 3 de Tanner) (Figure 1), une pilosité pubienne P1 faite d'un fin duvet et une pilosité axillaire absente A0. Il n'y avait pas de masses palpables au niveau inguinal. Les organes génitaux externes sont typiquement féminins avec présence des grandes et petites lèvres, pas de clitoromégalie, pas de fusion postérieure des grandes lèvres ni de gonades palpables au niveau des lèvres. On avait bien identifié deux orifices distincts: un orifice urétral et un autre vaginal. L'examen du vagin a montré un vagin perméable, de 8 cm de profondeur, d'aspect normal à la vaginoscopie, mais borgne. Au toucher rectal, l'utérus n'est pas palpable. Une échographie et une IRM abdomino-pelviennes ont montré l'absence de l'utérus et des ovaires. En plus, l'IRM a objectivé la présence de deux formations oblongues bilatérales de 44 mm de grand axe, tissulaires en discret hypersignal T2 de siège rétro-péritonéales siégeant en regard des vaisseaux iliaques externes, bilatérales et associées à des formations kystiques polaires inferieures de 17 mm de grand axe (Figure 2).

**Figure 3:**
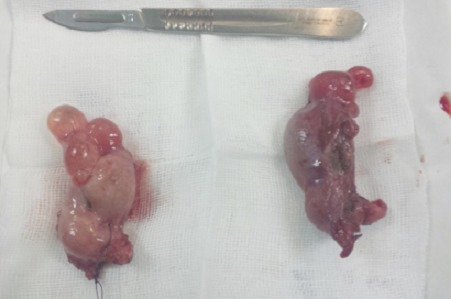
Testicules droit et gauche, renfermant sur leurs pôles inférieurs des formations kystiques

**Figure 4:**
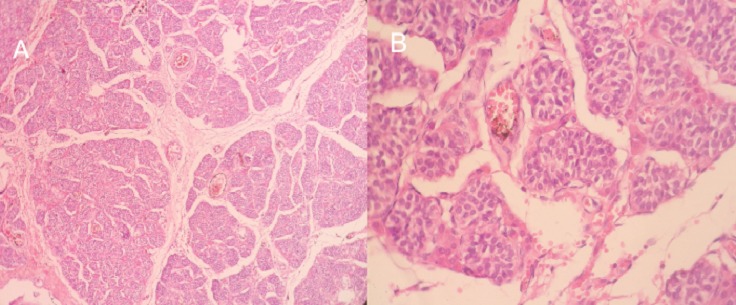
A) l’étude morphologique montre un parenchyme testiculaire d'architecture lobulée, fait de tubes séminifères d'aspect atrophique; B) ces tubes sont bordés de cellules de Sertolie, sans signes évident de spermatogenèse l'interstitium est fibreux comporte de rares amas de cellules de Leydig (responsable de sécrétion d'androgène)

Ces formations tissulaires arrivent au contact du pole supérieur de la vessie sans anomalie de cette dernière avec un canal vaginal en place. A la biologie elle présentait un taux de testostérone élevé 13,68 ng/ml dans les normes masculines, un taux de FSH bas à 1,2 UI/ l, un taus élevé de LH à 21 UI/l et un 17 bêta-œstradiol élevé à 58,6 pg/ml. Les marqueurs tumoraux (la βHCG était élevé à 114,5 mUI/ml, l'AFP et la LDH étaient normaux). Le caryotype a révélé une formule chromosomique 46, XY. Devant les antécédents de la patiente ainsi que ces constatations cliniques, biologiques et radiologiques, Le diagnostic du syndrome de résistance complète aux androgènes a été retenu. La patiente a bénéficie ainsi d'une orchidectomie bilatérale par laparotomie qui a permis la résection des deux testicules situés en rétro-péritonéal en regard des vaisseaux iliaques externes (Figure 3). A l'examen macroscopique, la gonade droite mesure 6×4×3 cm et la gonade gauche 7×4×3 cm renfermant sur leurs pôles inférieurs des kystes séreux. L'examen histologique a objectivé un parenchyme testiculaire immature fait de tubes séminifères souvent atrophiques d'aspect sertolien bordés par des cellules de Sertoli et parfois avec des très rares cellules de la lignée germinale sans spermatides ni spermatozoïdes visibles. Les tubes sont entourés par un tissu interstitiel tantôt fibreux tantôt lâche avec quelques cellules de Leydig par endroits et des remaniements kystiques sans signes histologiques de malignité (Figure 4). Un traitement à base d’œstro-progetaifs a été instauré en post opératoire. Après 6 mois de traitement hormonal substitutif, la patiente a maintenu un bon développement de ses caractères sexuels secondaires (développement mammaire stade 3 de Tanner).

### Observation 2

H.H est une jeune fille de 15 ans, fille unique, issue d'un mariage non consanguin. Elle a consulté à l’âge de 4 ans pour hernie inguinale bilatérale. Vu l'indisponibilité de l’ IRM à ce moment, une cœlioscopie a été réalisée qui a objectivé une migration incomplète des deux testicules au niveau de l'orifice profond de chaque canal inguinal avec absence de visualisation de l'utérus et des ovaires. Un caryotype réalisé a révélé une formule chromosomique 46 XY. La patiente n´a pas été traitée et il a été décidé d'attendre la puberté pour programmer une gonadectomie. La puberté est survenue chez cette patiente à l’âge de 13 ans avec un développement mammaire normal et un morphotype féminin harmonieux malgré l'aménorrhée. Elle pèse 70 kg et mesure 1,68 m soit un indice de masse corporelle à 24,80 kg /m^2^. La patiente a été admise à l'unité de gynécologie obstétrique pour une gonadectomie en période post pubertaire. L'examen physique retrouve un développement mammaire normal (stade 3 de Tanner) avec une aréole pale, une pilosité pubienne P1 faite d'un fin duvet et une pilosité axillaire absente A0. Les organes génitaux externes sont de type féminin sans ambiguïté: les grandes et petites lèvres sont présentes, sans clitoromégalie, les orifices urétral et vaginal sont distincts et un vagin perméable de 4 cm mais borgne. L'examen abdominal retrouve deux tuméfactions inguinales bilatérales. Une échographie pelvienne ainsi qu'une IRM pelvienne réalisées retrouvent l'absence d'organes génitaux internes (utérus et ovaires) et la présence de formations inguinales bilatérales faisant 46×14 mm à droite et 46×28 mm à gauche évoquant des testicules avec présence d'un nodule faisant 15×10mm au niveau du pôle supérieur du testicule gauche bien limité. Le bilan biologique retrouve un taux élevé de testostérone à 2,34 ng/ml mais qui était dans les normes masculines, un taux de FSH et de LH normaux respectivement de 4,3 mUI/ml et 4,72 mUI/ml, et un taux d’œstradiol à 26 pg/ml; les marqueurs tumoraux à savoir l'AFP, la LDH et βHCG étaient normaux.

**Figure 5:**
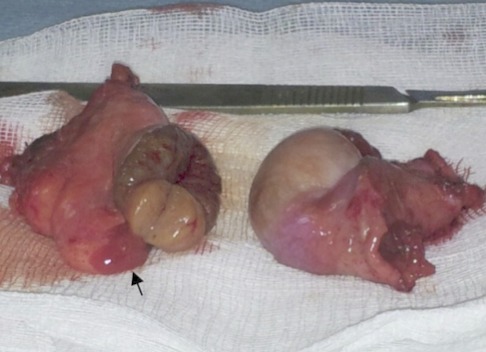
Testicules droit et gauche. Après une section du testicule gauche, un nodule se trouve à son pôle supérieur avec une formation à la périphérie, qui correspond à une ébauche de la trompe de Fallope (flèche)

**Figure 6:**
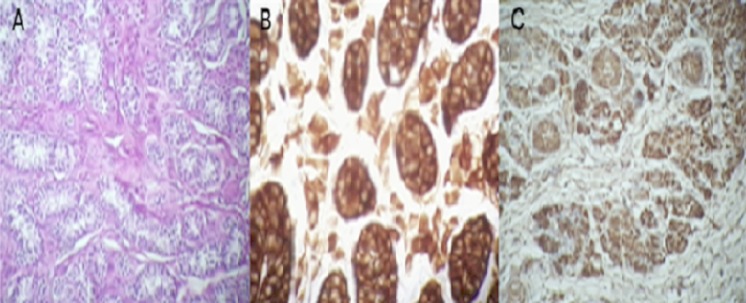
A) gonade atrophique composée principalement de tubules de cellules de Sertoli, avec absence de cellules germinales, et la présence de cellules interstitielles de Leydig, H&E x40; B) les cellules de Sertoli et les cellules de Leydig sont immunoréactives à l'inhibine x100; C) les cellules de Sertoli et les cellules de Leydig sont intensivement immunoréactive à Melan A x100

La patiente a bénéficié d'une orchidectomie bilatérale. A l'examen macroscopique, la gonade droite mesure 4×2×2 cm et la gonade gauche 4×3×2 cm renfermant sur la périphérie de son pole supérieur un nodule mesurant 1,3×1,3×1 cm (Figure 5). A l'examen histologique, le nodule bien limité, circonscrit par une fine capsule est composé de tubes sertoliens atrophiques avec un tissu interstitiel très réduit comportant de rares cellules de Leydig. Ce nodule, correspond à une tumeur bien différenciée à cellules de Sertoli-Leydig. A l'immunohistochimie, ces cellules expriment l'Inhibine et le Melan A (Figure 6). Le reste du parenchyme testiculaire atrophique est composé de cordons pleins remplis de cellules de Sertoli, sans spermatogonie. Le tissu interstitiel renferme des cellules de Leydig hyperplasiques associé à une ébauche de la trompe de Fallope sur la périphérie de la gonade gauche. Un traitement à base d’œstradiol a été instauré en post opératoire. Après 2 ans de traitement hormonal substitutif, la patiente a maintenu un bon développement de ses caractères sexuels secondaires (développement mammaire stade 3 de Tanner).

## Discussion

Le syndrome d'insensibilité complète aux androgènes est une entité rare; son incidence est en fait très variable, allant, selon les auteurs de 1/20000 à 1/60000 naissances [3]. Il est caractérisé par un déficit complet de l'action des androgènes au niveau des organes cibles lié à un dysfonctionnement du récepteur des androgènes en rapport avec une absence, ou un déficit, fonctionnel du récepteur cellulaire des androgènes, à un défaut qualitatif du récepteur, ou éventuellement, à un défaut touchant soit la transcription du gène, soit la liaison nucléaire du complexe stéroïde récepteur [4]. La conséquence est un morphotype typiquement féminin chez des sujets de caryotype masculin 46 XY. C´est une maladie héréditaire récessive liée au chromosome X, donc transmise par les femmes à une partie de leur descendance mâle. Cependant dans presque la moitié des cas, il s'agit d'une mutation de novo vu l'absence d'antécédents familiaux [5]. La recherche de la mutation causale s'effectue par séquençage du gène du récepteur des androgènes (RA). Les mutations au niveau du gène du RA sont très nombreuses, la substitution est l'anomalie la plus fréquente. Plus de 350 mutations responsables de syndrome de résistance aux androgènes sont répertoriées. Dans la majorité des cas, il s'agit d'une simple substitution d'acides aminés [5]. Au niveau de l'exon 1, Gottlieb et al. ont rapporté 65 mutations [6]. Concernant les mutations responsables d'un codon stop au niveau de l'exon 1, elles sont retrouvées dans 18 cas de formes complètes, dont 17 à type de substitutions et un seul cas de délétion. Bel Hadj et al. ont décrit une délétion de quatre bases de 1029 à 1032 de l'exon 1 du récepteur aux androgènes, occasionnant un codon stop en 476, et conduisant à une protéine tronquée non fonctionnelle [5]. Rarement, le diagnostic est établi en pré-pubertaire, à l´occasion de l´exploration d´une hernie inguinale comme c'est le cas de nos deux patientes. Dans la plupart des cas, les sujets consultent à l´adolescence ou après la puberté pour une aménorrhée primaire, voire une stérilité ou un syndrome tumoral à l’âge adulte.

L'examen physique retrouve souvent un développement morphologique féminin et harmonieux; une disposition gynoïde du tissu adipeux et une voix féminine; les seins sont bien développés et les cheveux sont normalement implantés, à l'opposé d'une absence de pilosité: les aisselles sont parfaitement glabres et le pubis ne présente aucun poil noir et dur. Ce signe doit attirer l'attention et faire évoquer le diagnostic. Cependant, un fin duvet clairsemé est possible et ne doit pas faire éliminer le diagnostic [7]. Il est classique de dire que les sujets atteints de ce syndrome se présentent comme de jolies femmes plus grandes que la moyenne féminine comme ce fut le cas de nos deux patientes et en général minces (le cas de notre première patiente) [5]. Les organes génitaux externes sont féminins avec un petit clitoris et des grandes et des petites lèvres bien développées, le vagin est petit et perméable se terminant en cul-de-sac. Les organes génitaux internes, dérivés des canaux de Wolff, sont souvent absents, il n'y a ni utérus, ni trompes, ni pavillon, ni prostate, mais la présence de résidus müllériens ne doit pas faire éliminer le diagnostic. La gonade est un testicule comportant un épididyme et un canal déférent; elle peut siéger en position inguinale, labiale ou abdominale [8]. Les résultats des dosages hormonaux doivent être interprétés en fonction des valeurs de références masculines et selon l’âge du patient. En période post pubertaire, le SICA est classiquement caractérisée par des taux de testostérone dans la zone masculine normale ou élevés vu la réponse exagérée à la stimulation par LH et des taux de LH sériques normaux ou élevés du fait de l'insensibilité hypothalamo-hypophysaire à la testostérone et des taux de FSH normaux [5]. L’échographie identifie aisément les testicules ectopiques lorsqu'ils sont dans les canaux inguinaux ou dans la région des grandes lèvres; sa performance est moindre lorsqu'ils sont en position abdomino-pelvienne. L'IRM pelvienne localise facilement les testicules ectopiques, qu'ils soient en intra-abdominal ou dans les canaux inguinaux ou les grandes lèvres; elle permet également de mettre en évidence de potentielles lésions focales en rapport avec une tumeur ou une inflammation et une éventuelle extension ganglionnaire [9]. La cœlioscopie comme ce fut le cas de l'une de nos patientes confirme l'absence d'utérus et d'ovaires. Le caryotype est masculin 46 XY. Histologiquement, le testicule du syndrome d'ISCA est formé de tubes séminifères immatures contenant de rares cellules germinales et bordés uniquement de cellules de Sertoli. Le nombre de cellules germinales peut être normal chez les enfants de moins de cinq ans et tend à diminuer voire s´annuler avec l´âge. Dans le tissu interstitiel, il existe une hyperplasie des cellules de Leydig associée dans 70% des cas [5]. Des ébauches de trompe de Fallope dérivant de l´extrémité des canaux de Müller sont présentes dans un tiers des cas en périphérie des testicules dû à un retard de synthèse de l'hormone anti-müllérienne par les cellules de Sertoli ou à son inactivation comme c’était le cas pour notre deuxième patiente.

Le principal risque est la cancérisation des testicules ectopiques. Ce risque est estimé à 5 - 10%, il est rare jusqu’à l’âge de 25 ans, puis augmente avec l’âge pour atteindre 33% après 50 ans [9]. Comme c´est le cas pour la cryptorchidie, la plupart des néoplasies chez les patientes atteintes de syndrome d'ISCA proviennent des cellules germinales [9]; en particulier le carcinome non invasif in situ, gonadoblastome et leurs homologues invasifs seminome/dysgerminome ainsi que les tumeurs non seminomateuses. Les tumeurs bénignes provenant des cellules non-germinales englobent les adénomes Sertoliens et les hamartomes [10]. La tumeur à cellules de Sertoli-Leydig est une tumeur rare du cordon sexuel et de stroma qui représente moins de 1% de l'ensemble des tumeurs ovariennes, survenant souvent à un âge jeune. Elle est également rarement développée chez le patient avec syndrome d'ISCA, il n'existe que deux cas rapportés dans la littérature [1]. Les tumeurs bénignes, bien différenciées représentent 10% de l'ensemble des tumeurs à cellules de Sertoli-Leydig et sont souvent associées au syndrome de testicule féminisant [11], c'est le cas de notre deuxième observation. Vu le risque de transformation maligne, l'orchidectomie bilatérale préventive doit être systématique par la laparotomie ou de préférence par coelioscopie lorsque les gonades sont de localisation intra-abdominale [12]. Elle est recommandée en période poste pubertaire afin de permettre un développement normal des organes génitaux externes et des seins durant la puberté quand la transformation maligne des cellules germinales est relativement rare et tardive [1]. L'orchidectomie peut également être pratiquée dans l'enfance quand le diagnostic est posé précocement lors d'une cure d'hernie inguinale. Cela, après une discussion des modalités thérapeutiques avec les parents qui peuvent choisir soit une gonadectomie avec induction de la puberté par des estrogènes ou de différer le geste en post pubertaire [10]. Pour nos deux patientes, la castration a été réalisée en post-pubertaire. La prise en charge du syndrome d'ISCA nécessite après la castration, l'instauration d'un traitement substitutif à base d’œstrogène, visant à prévenir les conséquences d'une ménopause par privation ostrogénique et à éviter la régression des caractères sexuels secondaires. Cette oestrogénothérapie peut être associée à un progestatif intermittent pour diminuer le risque de carcinogenèse mammaire [13]. Un soutien psychologique pourrait être également nécessaire aussi bien pour la patiente que pour sa famille surtout à l'annonce de la stérilité en évitant de révéler le sexe génétique.

## Conclusion

Le syndrome de résistance complète aux androgènes reste une entité rare. L'insensibilité des organes cibles aux androgènes est à l'origine du syndrome. Les tumeurs bénignes ont été décrites dans 80% des cas; elles sont représentées essentiellement par les adénomes sertoliens. Le risque de dégénérescence est assez faible mais augmente avec l’âge. Le traitement essentiel reste la castration pour éviter la dégénérescence gonadique et ce de préférence dès l’établissement du diagnostic en instaurant en pré-pubertaire une œstrogénothérapie substitutive pour assurer un développement harmonieux des caractères sexuels secondaires.
